# Core muscle rehabilitation during early mobilization after stroke: a narrative review and clinical framework

**DOI:** 10.3389/fneur.2026.1783386

**Published:** 2026-07-09

**Authors:** Chang-lan Ling-hu, Qin Yu, Meng Li, Zhen-zhen Liu, Xia-pei Peng

**Affiliations:** Department of Neurology, Wuhan Central Hospital, Tongji Medical College, Huazhong University of Science and Technology, Wuhan, Hubei Province, China

**Keywords:** balance, core muscle rehabilitation, early mobilization, early rehabilitation, falls, stroke, trunk training

## Abstract

**Objective:**

This narrative review clarifies the clinical framework for trunk- or core-focused rehabilitation after stroke, with particular attention to the early rehabilitation period. It distinguishes between preparatory bed-level exercise and true early mobilization.

**Methods:**

This narrative review selectively synthesizes recent reviews, consensus statements, randomized controlled trials, and cohort studies to inform clinical statements on early mobilization, trunk training, balance, falls, dysphagia, and clinical safety. Supplementary clinical reports are included primarily to illustrate practical exercise content and implementation patterns, rather than to form the primary basis for strong recommendations.

**Results:**

This review distinguishes upright early mobilization from preparatory bed-level trunk activation. Fixed hyperacute dose prescriptions are not supported by current evidence; core training is positioned as an adjunct to, rather than a substitute for, evidence-based functional training. Statements on falls and dysphagia are framed with greater caution. Table presents a pragmatic clinical decision-support framework, with acknowledged limitations in formal validation. Individualization according to age, stroke severity, stroke subtype, neurological status, and hemodynamic tolerance is consistently emphasized.

**Conclusion:**

Core muscle rehabilitation can be integrated into early stroke rehabilitation once patients achieve medical and neurological stability. The current evidence most strongly supports its efficacy for improving trunk control and balance; recommendations regarding dosage, direct fall prevention, and specific benefits for dysphagia should remain individualized and conservative.

## 1 Introduction

Stroke remains a leading cause of death and long-term disability globally, with contemporary analyses confirming its substantial burden across regions and age groups (1–5). Many survivors experience impaired trunk control, reduced balance, limited mobility, and non-motor complications that affect both safety and the pace of rehabilitation (6–9). The early post-stroke period represents a critical window for rehabilitation, though its terminology requires precision. Within current rehabilitation literature, “early mobilization” typically denotes upright or out-of-bed activity after medical stabilization, rather than bed-based exercise alone (10–12). Consequently, activities such as bridging and assisted rolling are more accurately framed as preparatory early rehabilitation, not as early mobilization per se. This narrative review examines trunk-focused rehabilitation after stroke and presents updated definitions, safety considerations, dosage statements, and conclusions that reflect contemporary evidence. Trunk-focused training serves as a potentially useful adjunct within a staged rehabilitation approach, but it should not be prioritized over established functional mobility training in the very early phase after stroke (2–5, 10–12).

## 2 Methods of literature search

This article is structured as a selective, non-systematic narrative review with a primary clinical focus. To examine the effects of core muscle exercises on trunk stability, postural control, and balance in early stroke rehabilitation, literature was reviewed selectively. The synthesis prioritizes recent high-level evidence, including randomized controlled trials, Cochrane reviews, cohort studies, and consensus statements. As a narrative review, this article does not follow a PRISMA-guided systematic search, nor does it include a quantitative synthesis. Studies were selected subjectively based on their clinical relevance to early mobility, trunk training, and post-stroke complications.

Furthermore, this review integrates practical “clinical experience,” which specifically refers to the routine care pathways and expert consensus developed by the multidisciplinary neurorehabilitation team at our institution. This institutional experience was incorporated solely to illustrate pragmatic exercise content and to organize the clinical decision-support framework (Table 1), rather than to serve as empirical evidence. We acknowledge that the narrative design and the integration of local institutional practices inherently introduce selection and subjective biases.

**Table 1:** Proposed clinical considerations before initiating trunk-focused rehabilitation in the early rehabilitation window after stroke.

System / Domain	Clinical considerations / Exclusion concerns
Neurological	Neurological deficit should be clinically stable; No evidence of uncontrolled intracranial hypertension; Adequate arousal and ability to participate (for example, GCS ≥ 13 when applicable).
Cardiovascular	Hemodynamic stability without uncontrolled arrhythmia, active myocardial ischemia, or decompensated heart failure; Blood pressure and heart-rate responses should be acceptable for the planned activity level.
Other medical issues	No untreated deep venous thrombosis. No uncontrolled epileptic seizures. No severe sepsis, or other acute medical problem that would make upright or resisted activity unsafe.
Patient factors	Recommendations should be individualized according to: Age, stroke severity, stroke subtype. Level of consciousness, cardiopulmonary reserve. Orthostatic tolerance, fatigue, and coexisting complications.

These items are pragmatic clinical checkpoints for decision-making rather than prospectively validated trunk-training thresholds.

## 3 Overview of core muscle rehabilitation

The core muscle system comprises the lumbopelvic-hip complex, which provides proximal stability, transmits force, maintains postural alignment, and regulates movement. While historical models of spinal stability and neuromuscular facilitation informed the modern concept of core control, current clinical understanding also incorporates anticipatory postural adjustments, respiratory control, dynamic trunk coordination, and task-specific postural performance (13–16). In stroke rehabilitation, trunk function is clinically significant as it underpins sitting balance, transfers, weight shifting, standing, and gait training. Systematic reviews and meta-analyses indicate that trunk- or core-focused training may be associated with improvements in trunk function and certain balance outcomes, especially when combined with conventional physiotherapy, although the certainty of evidence is constrained by study heterogeneity, co-interventions, and variations in stroke phase and intervention content (17–24).

## 4 The early mobilization period: concept and risks

### 4.1 Conceptual clarification

Although no universally accepted single definition of the “early” rehabilitation period after stroke exists, establishing conceptual clarity is essential to avoid methodological ambiguity. We adopt a dual framework: the landmark Stroke Recovery and Rehabilitation Roundtable (SRRR) consensus defines the overarching timeline of stroke recovery (17), while the seminal AVERT trial informs the categorization of specific mobilization protocols (18).

Based on the SRRR consensus, the post-stroke physiological timeline is defined as:

- Acute Phase: The first 1 to 7 days post-stroke.
- Subacute Phase: From 7 days to 6 months post-stroke.
- Within these temporal boundaries, clinical mobilization is categorized as follows:
- Early Rehabilitation: A broad umbrella term covering the entire acute phase, beginning once the patient is medically and neurologically stable. It encompasses both preparatory bed-level exercises and progressive out-of-bed activities.
- Very Early Mobilization (VEM): As strictly defined by the AVERT trial, this refers to upright, out-of-bed activity initiated within the first 24 h post-stroke.
- Early Mobilization: Upright physiological challenges (e.g., supported sitting, standing, walking) initiated between 24 and 48 h post-stroke and extending through the first week.

This review focuses on the transition from the acute to the early subacute phase. For clinical and methodological precision, we use a staged progression. Within the broader “early rehabilitation” window, the term “early mobilization” is strictly reserved for true out-of-bed and upright activities. It must be distinguished from preparatory bed-level trunk activation, as their physiological impacts and underlying evidence bases differ markedly.

Furthermore, the integration of core-specific training currently lacks a globally standardized temporal definition. To avoid conflating these categories, early core training is conceptualized as an adjunctive, preparatory component initiated within the acute phase. Core training builds foundational trunk stability to support true upright mobilization once the patient is medically cleared, without delaying it (25–29).

### 4.2 Risk factors and safety considerations

Safety remains the primary concern during this phase. Following a stroke, patients often present with weakness, impaired trunk control, sensory loss, visuospatial deficits, dysphagia, cognitive impairment, cardiac instability, or fluctuating blood pressure, all of which affect the risks associated with mobilization. Prolonged bed rest also contributes to deconditioning, thrombosis, infection, contracture, and a loss of functional reserve (8, 9, 30–38). Recent studies confirm that serious falls and fall-related fractures are significant clinical problems after stroke, though these outcomes are multifactorial and are typically reported over longer follow-up periods rather than in the initial days post-stroke (30–32). Trunk-focused rehabilitation may improve risk markers for falls, such as balance and transfer quality, though direct evidence for reducing early fall incidence remains limited.

## 5 Clinical implementation framework

### 5.1 Timing and dose of intervention

Structuring intervention timing according to a patient’s functional stage remains a highly practical clinical approach. Current evidence does not support rigid, fixed-dose core training protocols, particularly during the hyperacute and early acute phases. Shorter and more frequent supervised rehabilitation sessions are generally preferable in the initial days post-stroke. The optimal timing, intensity, and specific content of these sessions must be meticulously individualized rather than dictated by a universal protocol (10–12, 19–24).

Once neurological and hemodynamic stabilization is achieved, preparatory bed-level interventions such as trunk activation, assisted rolling, pelvic control, bridging, and supported midline exercises can be safely initiated. These preparatory activities serve as adjuncts to higher-level functional mobility and should not replace it. When clinically safe and physiologically feasible, upright activities such as standing, transferring, and gait-related practice must take precedence. The overarching clinical priority remains early, evidence-based functional mobilization, strictly tailored to the individual patient’s cardiopulmonary tolerance, stroke severity, and functional status.

### 5.2 Patient eligibility and individualization

In the absence of a universally established or standardized framework specifically for early core muscle rehabilitation, Table 1 presents a pragmatic clinical framework synthesized from general acute stroke mobilization guidelines and expert consensus (33). It organizes bedside decision-making and highlights common exclusion concerns. These recommendations require individualization based on age, stroke severity and subtype, neurological stability, level of consciousness, cardiopulmonary reserve, hemodynamic tolerance, and coexisting complications.

### 5.3 Exercise prescription and positioning within rehabilitation

Current evidence describes a wide range of interventions targeting the trunk and core, from bed-level foundational exercises (e.g., abdominal bracing, pelvic tilting, bridging, and assisted rolling) to progressive upright activities (e.g., seated weight shifts, lateral reaching, trunk rotation control, and dynamic balance tasks). Although some trials have examined isolated trunk training, most studies embed these exercises within multimodal regimens that combine conventional physiotherapy, task-oriented practice, virtual reality, automated sitting-balance technologies, or neuromodulation (19–24, 39–46). Because most positive functional outcomes arise from these comprehensive programs, isolating the specific efficacy of core training alone remains methodologically difficult. Accordingly, isolated core exercises should not replace established, evidence-based functional mobility training during the early acute phase. A more evidence-aligned view is that trunk-focused rehabilitation functions best as a synergistic, adjunctive component, seamlessly integrated into the broader functional progression of sitting, transferring, standing, and gait training (2–5, 19–24, 39–46). Moreover, treatment dosages reported in the literature vary considerably. Recent trials and reviews document session durations from 20 to 60 min, frequencies ranging from several times per week to daily supervised practice, and total intervention periods of 4 to 8 weeks. This substantial variance highlights the absence of a standardized early prescription and reinforces the need for a cautious, highly individualized dosing approach rather than adherence to a rigid, universal protocol (19–24, 47–56).

### 5.4 Expected outcomes and evidence synthesis

Based on the available evidence, the most consistently reported short-term benefits of trunk-focused interventions after stroke include improved trunk control, dynamic sitting balance, and postural alignment. Although downstream functional gains—such as those in gait parameters, activities of daily living (ADLs), and quality of life—are frequently observed, these outcomes show greater variability and are highly susceptible to confounding by concurrent therapies (19–24, 39–46). Stratification of the literature by study design and stroke phase (Table 2) reveals that the most robust, high-certainty evidence comes primarily from subacute or chronic stroke cohorts, rather than from hyperacute or early acute stages. The positive outcomes reported in comprehensive, multimodal rehabilitation programs should be interpreted with caution, as these benefits reflect the combined effect of a synergistic treatment package and cannot be attributed solely to the isolated causal effect of the core-training component.

**Table 2:** Summary of Representative High-Level Evidence and Clinical Studies on Post-Stroke Early Mobilization and Trunk Rehabilitation.

Author & Year	Country	Study design	Sample size	Stroke phase	Intervention focus	Comparator	Dose / Duration	Main outcomes / Key findings	Main limitations	Key message for the present review
Miranda et al. (10)	Brazil	Systematic Review	7 trials (*N* = 863)	Acute	Early mobilization (EM, out-of-bed activity)	Routine care	15–45 min, 1–3 times/day	Improved functional capacity (mRS). Optimal start time identified as >24 h post-stroke.	Did not fully standardize EM protocols across all included studies.	Supports revised terminology and strictly distinguishes EM from bed-level preparatory exercises.
Ding &Zhang (11)	China	Meta-analysis	8 RCTs	Acute	Very early mobilization (VEM)	Standard care	Various VEM intensities	Shortened hospital stay and improved ADL without increasing adverse events.	Lack of standardized dosing; potential performance bias in primary trials.	Supports individualized timing and mandates caution regarding fixed, aggressive early dosages.
Thijs et al. (19)	Belgium	Cochrane Systematic Review	68 trials (*N* = 2,585)	Across stroke phases	Trunk training (core stability, selective/unstable)	Standard therapy / non-dose-matched	Various (average or non-equal duration)	Significant benefits for ADL, trunk function, standing balance, and walking ability.	Very low to moderate certainty of evidence; small sample sizes in most trials.	Confirms broad benefits for trunk and balance outcomes, though high-certainty evidence remains limited.
Zheng et al. (21)	China	Network Meta-analysis	Multiple RCTs	Across stroke phases	Core stability training (CST)-based combination therapies	CST alone or conventional PT	Evaluated across multiple durations	AT+CST and PT + CST identified as optimal combinations for lower extremity dysfunction.	Indirect comparisons inherent to NMA; clinical heterogeneity.	Provides recent highest-level evidence that combining core training significantly optimizes motor recovery.
Rayner et al. (34)	UK	Systematic Review &Meta-analysis	16 trials (*N* = 623)	Early - acute	Targeted physiotherapy sitting balance treatments	Standard care	Focused on early sub-acute phase dosages	Significant improvements in sitting balance primary outcome (Trunk Impairment Scale).	Some included studies showed concerns regarding risk of bias.	Confirms the effectiveness of targeted physiotherapy specifically for sitting balance in the early sub-acute phase.
Cabana-Valdés et al. (22)	Spain	Randomized Controlled Trial (RCT)	*N* = 87	Early sub-acute (≤30 days)	Additional core stability exercises (CSE) + CP	Conventional physiotherapy (CP)	5 days/week for 5 weeks	Improved dynamic sitting balance, trunk coordination, lower-limb spasticity, and balance.	Single-center design; specific to early sub-acute rather than hyperacute phase.	Demonstrates targeted efficacy but emphasizes findings are not directly generalizable to the hyperacute period.
Van Criekinge et al. (25)	Intl.	Expert Consensus Statement	13 worldwide experts	All phases	Balance and mobility measurement standards	N/A	N/A	Established a core set of standardized balance assessments (e.g., TIS, Mini-BESTest, BBS).	Subjective bias inherent to expert consensus panels.	Crucial for guiding standardized outcome selection and measurement interpretation in early rehab.
Wang et al. (31)	Intl. (5 countries)	Large Rehabilitation Database Study	*N* = 2,104	Early post-stroke	Epidemiological tracking of serious falls	N/A	Observation over 12 months	4% experienced serious falls; impaired early mobility strongly associated with heightened fall risk.	Excluded individuals with poor pre-morbid mobility; potential underestimation.	Highlights that early falls remain critically important, reinforcing the need for early core stabilization.

## 6 Critical appraisal and extended considerations

### 6.1 Balance, function, and fall risk

From a rehabilitation standpoint, gains in trunk control and postural alignment carry clinical significance by enabling safer transfers, improving sitting and standing balance, and supporting more effective gait training. Although it is physiologically reasonable that such improvements may lower fall risk, current evidence in early stroke rehabilitation primarily reflects enhancements in surrogate measures such as Berg Balance Scale scores, trunk impairment scales, and movement quality, rather than a documented reduction in actual fall incidence (25–32, 39–46). Core-focused rehabilitation serves as a modifying factor that enhances balance and stability, though direct evidence for preventing early post-stroke falls remains limited.

### 6.2 Swallowing and breathing considerations

Post-stroke dysphagia remains a major complication that necessitates early screening, multidisciplinary assessment, and targeted management. Current guidelines and reviews highlight structured screening, instrumental assessment where indicated, dietary modification, oral care, and specific dysphagia rehabilitation as the cornerstone of care (33–38).

While posture and respiratory mechanics can influence swallowing, there is no direct evidence that isolated core training alters swallowing or respiratory outcomes. Therefore, these elements should be presented only as potential or associated considerations, and trunk optimization is framed merely as a potentially supportive postural component within a more comprehensive rehabilitation program.

### 6.3 Integration with other modalities

Interest in combined rehabilitation strategies remains high, with recent reviews and trials describing potential additive effects when trunk- or balance-focused training is integrated with neuromuscular electrical stimulation, robot-assisted gait training, non-invasive brain stimulation, virtual reality, or other feedback-rich approaches (39–46, 57–64). These multimodal studies must nevertheless be interpreted carefully. While they illustrate feasible implementation pathways and suggest future directions, they do not justify attributing all observed gains to the core training component itself.

Furthermore, since trunk exercises are almost always embedded in multimodal rehabilitation programs, isolating the specific causal contribution of core muscle training presents a methodological challenge. Observed gains should therefore be interpreted as outcomes associated with a comprehensive rehabilitation package rather than definitive effects of core training alone.

### 6.4 Limitations of the current evidence and this review

Several methodological limitations concerning both the existing evidence base and the design of this review merit acknowledgment.

First, as a narrative review, the literature search was intentionally non-exhaustive, so the possibility of selection bias cannot be entirely excluded. Correspondingly, we did not perform a formal risk-of-bias assessment (e.g., Cochrane RoB 2 or AMSTAR) across the included studies.

Second, the synthesized primary literature is constrained by several inherent methodological shortcomings. The current evidence base consists largely of studies with small sample sizes and considerable heterogeneity, which spans both patient demographics (differing stroke severities and subtypes) and intervention parameters (dosage, frequency, and specific core exercises). Moreover, critical appraisal reveals that many cited trials exhibit prevalent limitations, including unclear allocation concealment and a lack of blinding—though the latter is a well-recognized, inherent challenge in physical rehabilitation trials. Collectively, these factors introduce potential performance bias and limit the external validity and generalizability of our conclusions.

Finally, the practical framework and clinical care pathways proposed (including Table 1) are heavily informed by the specific clinical experiences and protocols of a single institution. While this offers a pragmatic clinical blueprint, it inevitably introduces subjective bias. Accordingly, this framework may not be universally applicable across all clinical settings and should be locally adapted based on specific healthcare resources and patient populations.

Given these limitations, our findings should be interpreted with caution. The broader application of early core training to all stroke survivors remains a promising but still-evolving paradigm, one that ultimately requires substantiation through larger, high-quality, rigorously controlled multicenter trials.

## 7 Conclusion

Stroke remains a leading global cause of long-term neurological disability and poses a formidable public health challenge. The early post-stroke mobilization phase, functionally defined as the critical transition from bed rest to upright standing, represents a pivotal therapeutic window for neurorehabilitation. Given the high prevalence of trunk instability after stroke and its foundational role in postural control and balance, core muscle rehabilitation is a clinically logical component of early stroke care and is essential for facilitating progressive mobility (1–12, 19–25).

However, the clinical implementation of trunk-focused interventions must be rigorously evidence-based. Once neurological and hemodynamic stability is achieved, core rehabilitation should be highly individualized, phase-appropriate, and seamlessly integrated into broader, task-oriented functional training. According to current literature, the strongest evidence supports the efficacy of these interventions for improving local trunk control and dynamic balance. Conversely, proposing direct causal benefits of isolated core training for absolute fall prevention, specific improvement of dysphagia, or the necessity of fixed hyperacute dosing regimens remains premature and warrants further rigorous investigation (19–46).
